# Levetiracetam treatment ameliorates LRRK2 pathological mutant phenotype

**DOI:** 10.1111/jcmm.14674

**Published:** 2019-09-27

**Authors:** Mauro Rassu, Alice Biosa, Manuela Galioto, Milena Fais, Paola Sini, Elisa Greggio, Giovanni Piccoli, Claudia Crosio, Ciro Iaccarino

**Affiliations:** ^1^ Department of Biomedical Sciences University of Sassari Sassari Italy; ^2^ Department of Biology University of Padova Padova Italy; ^3^ Department of Cellular, Computational and Integrative Biology University of Trento Trento Italy

**Keywords:** Levetiracetam, LRRK2, Parkinson's disease, SV2A

## Abstract

Mutations in leucine‐rich repeat kinase 2 (LRRK2) are the most common genetic cause of Parkinson's disease (PD). The LRRK2 physiological and pathological function is still debated. However, different experimental evidence based on LRRK2 cellular localization and LRRK2 protein interactors suggests that LRRK2 may be part and regulate a protein network modulating vesicle dynamics/trafficking. Interestingly, the synaptic vesicle protein SV2A is part of this protein complex. Importantly, SV2A is the binding site of the levetiracetam (LEV), a compound largely used in human therapy for epilepsy treatment. The binding of LEV to SV2A reduces the neuronal firing by the modulation of vesicle trafficking although by an unclear molecular mechanism. In this short communication, we have analysed the interaction between the LRRK2 and SV2A pathways by LEV treatment. Interestingly, LEV significantly counteracts the effect of LRRK2 G2019S pathological mutant expression in three different cellular experimental models. Our data strongly suggest that LEV treatment may have a neuroprotective effect on LRRK2 pathological mutant toxicity and that LEV repositioning could be a viable compound for PD treatment.

## 1 INTRODUCTION

Approved by the US Food and Drug Administration in the 1999, levetiracetam (LEV) is a widely used drug for the treatment of partial and generalized epilepsy. In contrast to the common antiepileptic compounds, which preferentially bind to voltage‐gated sodium channels in their inactive conformation, LEV binds the integral transmembrane protein SV2A, localized on both synaptic dense‐core vesicles and small clear vesicles containing neurotransmitters and almost ubiquitously expressed in the different areas of central nervous system (CNS).^1^ Up to date, although the clear involvement in seizure control, the SV2A physiological role is far from being understood. Among the functions proposed, calcium‐dependent exocytosis, neurotransmitter loading/retention in synaptic vesicles, synaptic vesicle priming and transport of vesicle constituents are the most robust (for review, see Ref.^2^). A possible molecular mechanism by which SV2A controls vesicle dynamics is via the interaction with synaptotagmin 1.^3^ The SV2A role in the seizure control is further confirmed by the analysis of SV2A knockout mice. A large proportion of SV2A knockout mice die immediately after birth and the surviving knockout soon develop frequent seizures that lead to premature death.^4^ Primary neurons from SV2A knockout mice present synapses that are ultrastructurally indistinguishable from the wild‐type mice.^4^ Interestingly, alteration in vesicle trafficking seems a common feature shared by different PD causative genes.^5^ Mutations in the leucine‐rich repeat kinase 2 gene (LRRK2, PARK8) are the most frequent genetic causes of Parkinson's disease, reaching up to 40% in some ethnic groups, that is Ashkenazi Jewish and North African Arab Berbers.^6^ Although LRRK2 physiological and pathological function is largely debated, different lines of evidence have pointed out an important role of LRRK2 in the control of vesicle trafficking that in turn may explain the different cellular dysfunctions ascribed to mutant LRRK2 expression.^7^, ^8^, ^9^

Interestingly, Marcotulli and colleagues have analysed the effect of LEV on presynaptic proteins level and distribution in the rat cerebral cortex.^10^ In this experimental model, the expression of different vesicular proteins is modified upon LEV treatment and LRRK2 is part of this protein network.^10^ In addition, SV2A was pulled down from adult mouse brain lysates by the LRRK2 WD40 domain^11^ suggesting a their involvement to common cellular pathways.

Based on the previous considerations, it is reasonable to speculate that SV2A and LRRK2 are both involved in the modulation of vesicle dynamics, although the molecular mechanisms are still unknown. Whether LRRK2 and SV2A have a functional interaction and more important whether they have an opposite or synergistic biological effect remain to be explored. To test this hypothesis, we have evaluated the ability of LEV to affect the LRRK2 cellular effects in different experimental models.

## 2 MATERIAL AND METHODS

### 2.1 Primary cortical neurons and neurite measurement

Experimental procedures involving the use of animals were approved by the Italian Ministry of Health (licence 318/2013‐B and licence 1041/2016‐PR). C57BL/6 LRRK2 wild‐type (WT) and LRRK2 G2019S BAC mice were obtained from Jackson Laboratory [B6.Cg‐Tg(Lrrk2*G2019S)2Yue/J]. Housing and handling of mice were done in compliance with national guidelines. Primary neurons were prepared from brain cortex as previously described.^7^ Neurons were transfected with 1 µg of GFP plasmid at 3 days in vitro (DIV) using Lipofectamine 2000. Starting from DIV3, neurons were treated with 5 µmol/L LEV every 48 hours, fixed in 4% paraformaldehyde at DIV 7 and then processed for immunofluorescence. The mouse anti‐tubulin III (clone 2G10, 1:500 Sigma) was used as a neuronal marker. Fluorescent images of GFP‐positive neurons were acquired using the epifluorescent microscope Leica 5000B with a 40× objective. Exposure settings for the GFP channel were kept constant across images and experiments. Each microscope field includes one single neuron. The images were taken by a blinded operator, and the total sum of all traced neurites was performed on GFP images using NeuronJ with a total of ≈ 20 neurons analysed/genotype/condition. Any type of GFP‐transfected cortical neurons was selected for the analysis.

### 2.2 PC12‐G2019S differentiation and analysis

PC12 cells stably expressing doxycycline (dox) inducible LRRK2 G2019S mutant^12^ were grown in DMEM–F12, 10% Fetal Bovine Serum (FBS) at 37°C. For differentiation experiment, the cells were plated at low density (5% of confluence) on a cover glass, previously treated with poly‐L‐lysine solution for 1 hour. The differentiation was performed growing the cells in 1% FBS in the presence of 100 ng/mL of NGF for 6 consecutive days, adding new medium containing NGF each 48 hours. Doxycycline (0.2 μmol/L) and LEV (10 μmol/L) were added together with NGF and replaced every 48 hours in the same experimental conditions. At the end of the experimental procedure, the differentiated cells were fixed with 4% paraformaldehyde/PBS and analysed by phase‐contrast microscopy. For Western blot analysis, the cells were plated on 24 multiwell culture plates in the same experimental conditions. The cells were lysed by Laemmli buffer, and the protein extracts were normalized by histone 4 (H4) or beta‐actin levels.

### 2.3 SH‐SY5Y transduction, immunofluorescence and Western blot

SH‐SY5Y cells stably expressing dopamine receptor D2 carrying a Flag epitope (SH‐SY5Y‐DRD2)^7^ were grown in DMEM–F12, 10% Fetal Bovine Serum (FBS) at 37°C. Cell transduction and immunofluorescence experiments were performed as previously published.^7^ Briefly, the cells were grown on a cover glass and transduced by adenoviral particles (10‐30 pfu/cell) in DMEM‐F12. 48 hours after transduction, the cells were fixed with 4% paraformaldehyde/PBS and permeabilized with 0.1% Triton X‐100. Cells were incubated with primary antibodies: anti‐LRRK2 (1:500 Mjff2 c41‐2 epitomics), anti‐Flag (1:2500 Sigma‐Aldrich) and anti‐SV2A (HPA007863 1:500 Sigma‐Aldrich) and then incubated with secondary antibodies: Goat Secondary Antibody Alexa Fluor® 488 or Alexa Fluor^®^ 647 (Life Technologies). The cells were analysed by Leica TCS SP5 confocal microscope with LAS lite 170 image software.

Western blot analysis was performed as previously described.^7^ Briefly, equal amounts of protein extracts were resolved by standard sodium dodecyl sulphate‐polyacrylamide gel electrophoresis. Samples were electroblotted onto Protran nitrocellulose (GE Healthcare Life Sciences). Membranes were incubated with 3% low‐fat milk in 1X PBS‐Tween 0.05% solution with the indicated antibody: anti‐Myc (clone 9E10 1:5000 Sigma‐Aldrich), anti‐Flag (F3165 1:2500 Sigma‐Aldrich), anti–beta‐actin (A5441 1:5000 Sigma‐Aldrich), anti‐histone H4 (SAB4500313 1:1000 Sigma‐Aldrich), anti–β3‐Tubulin (4466 1:1000 Cell Signaling), anti‐SV2A (HPA007863 1:1000 Sigma‐Aldrich) for 16 hours at 4°C. Goat antimouse immunoglobulin G (IgG) peroxidase‐conjugated antibody (1:2500 Millipore Corporation) or goat anti‐rabbit IgG peroxidase‐conjugated antibody (1:5000 Millipore Corporation) were used to reveal immunocomplexes by enhanced chemiluminescence (Pierce Biotechnology).

### 2.4 Statistical analysis

The results are presented as means ± SD of at least n = ≥3 independent cultures. Statistical evaluation was conducted by one‐way ANOVA test and Bonferroni's post‐test or Student's *t* test as indicated. Values significantly different from the relative control are indicated with an asterisk. *P < .05; **P < .01; ***P < .001.

## 3 RESULTS

### 3.1 Effect of LEV on the inhibition of neurite outgrowth due to LRRK2 G2019S expression in primary neurons and in PC12 cells

**Figure 1:**
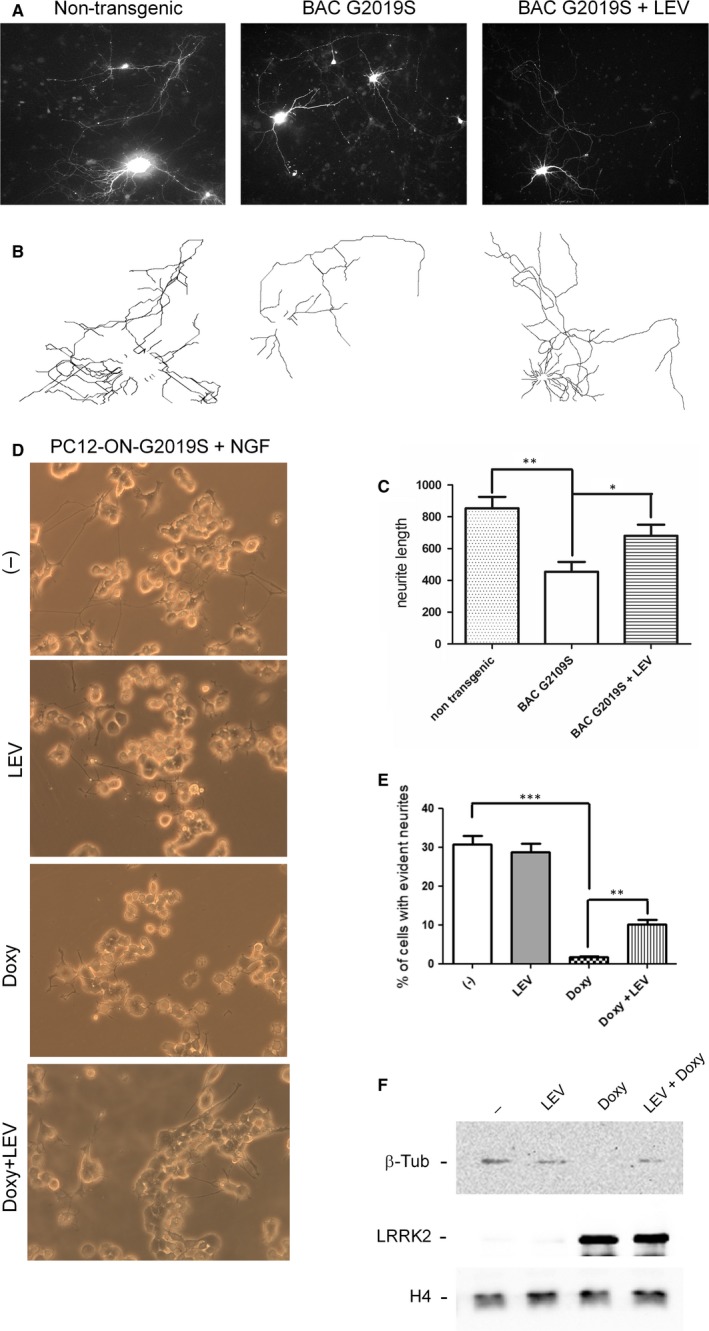
Analysis of LEV effect on neurite branching on primary cortical neurons or PC12 cells expressing LRRK2 G2019S. A‐B, Primary neurons from BAC G2019S LRRK2 transgenic animals were transfected at DIV3 with GFP and then treated with 5 μmol/L LEV from DIV3 to DIV7. At DIV7, the cells were fixed and the neurite extension was measured analysing the GFP expression by confocal microscope (B). C, Quantification of data obtained in (B). The data represent the sum of total length of neurites/neuron in two independent replicates and are represented as mean ± SD. *P < .05; **P < .01. One‐way ANOVA test and Bonferroni's post‐test were used. D, PC12 cells stably expressing dox‐inducible LRRK2 G2019S were treated for 6 d with NGF in the presence or absence of dox or dox + LEV. E, Quantification of data obtained in (D). The data represent the numbers of cells clearly showing neurite extensions in three independent replicates and are represented as mean ± SD. **P < .01; ***P < .001. One‐way ANOVA test and Bonferroni's post‐test were used. F, PC12‐G2019S cells treated as previously described were lysed and analysed by Western blot using specific antibodies for the indicated proteins (anti–β3‐tubulin, anti‐Myc antibody for LRRK2). Histone H4 serves as controls for equal number of cells

**Figure 2:**
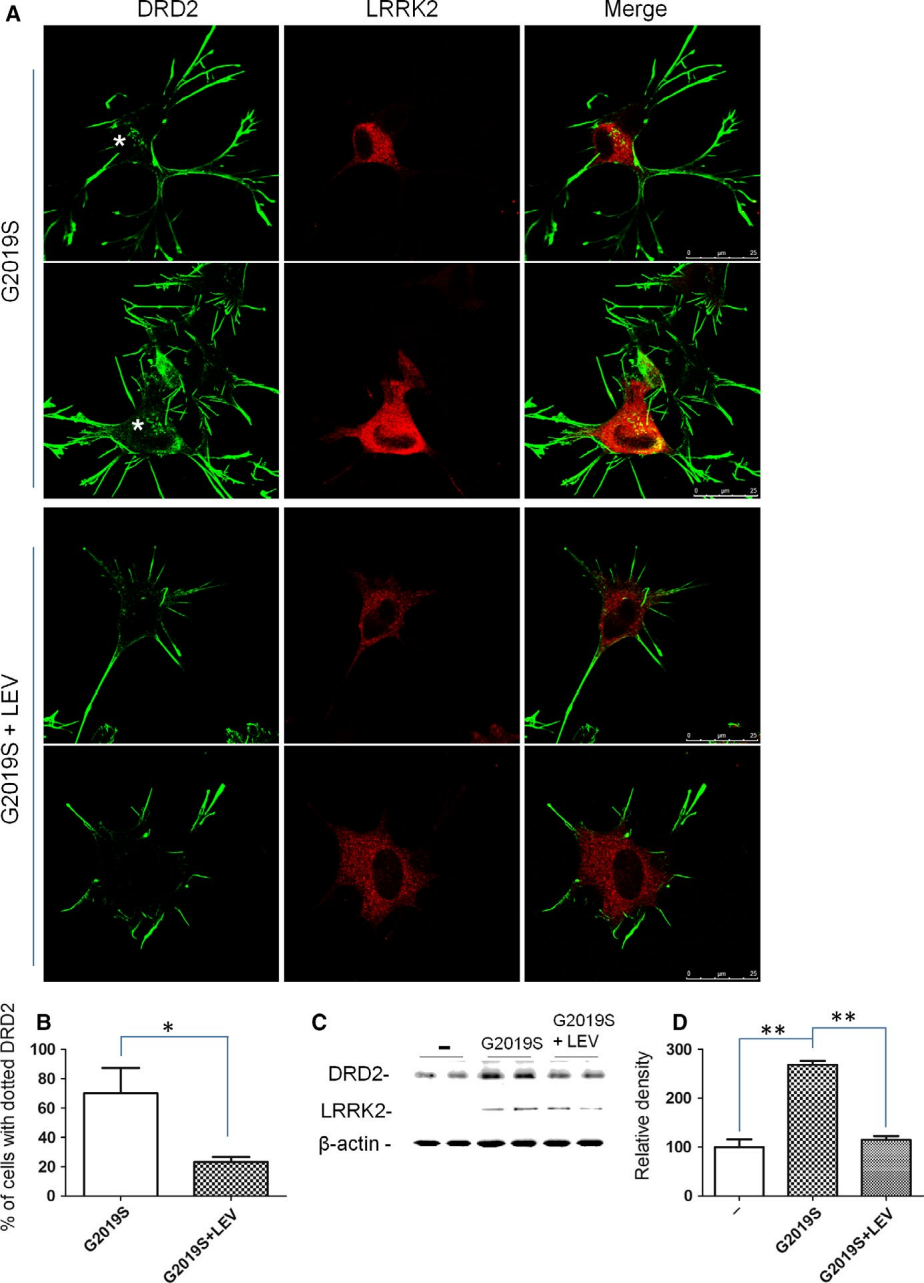
Analysis of LEV effect on DRD2 localization and level in the presence of LRRK2 G2019S. A, SH‐SY5Y cells stably expressing Flag‐tagged DRD2 were transduced by LRRK2 G2019S and treated or not for 48 h with LEV. The cells were fixed and incubated with different primary antibodies (anti‐Flag for DRD2 and anti‐LRRK2 antibody). The asterisk indicates the DRD2 in the Golgi areas. Scale bars = 25 µm. B, Quantification of data obtained in (A). The data represent the mean ± SD. *P < .05; Student's *t* test was used. C, Cells treated as previously described were lysed and analysed by Western blot using specific antibodies for the indicated proteins (anti‐Flag for DRD2 and anti‐Myc antibody for LRRK2). β‐actin serves as controls for equal loading of samples. D, Quantification of data obtained in (C). The data represent the mean ± SD; **P < .01. One‐way ANOVA test and Bonferroni's post‐test were used

Primary neurons from WT or LRRK2 G2019S BAC transgenic mice were prepared and transfected with GFP to fill the cell and trace the neurite branching and length. The LEV concentration to be used in the assay was established by MTS assays. At 5 μmol/L of LEV, no significant toxic effect was detected (data not shown). At DIV3 and DIV5, the primary neurons were treated with 5 μmol/L LEV and subsequently analysed at DIV7. As shown in Figures 1, 2A‐B‐C, the G2019S expression determines a significant reduction in neurite length compared with WT primary neurons, as previously reported.^7^ Of note, LEV treatment significantly ameliorates the neurite shortening phenotype of G2019S neurons (Figures 1, 2A‐B‐C).

To confirm the data obtained on primary neurons, we used a different experimental model: PC12 cells expressing the dox‐inducible G2019S LRRK2 mutant.^12^ The cells were treated with NGF to induce neuronal differentiation in the presence or absence of dox. As illustrated in Figure 1D‐E, roughly 30% of PC12‐G2019S show significant neurite outgrowth after 6 days of NGF treatment. The concomitant expression of G2019S mutant, by doxycycline treatment, determines a strong reduction in neurite outgrowth (roughly 1%‐2% of cells). The LEV addition partially, but significantly, increases the neurite outgrowth (roughly 10% of cells) (Figure 1D‐E). No effect of LEV was visible in PC12‐G2019S cells without dox treatment. To further validate the differentiation process, we used the β3‐tubulin marker. In a preliminary experiment, we have evaluated the β3‐tubulin expression level in PC12‐G2019S cells. The β3‐tubulin protein was almost undetectable in our experimental conditions (data not shown). Then, we performed the same differentiation protocol, and at the end of the procedure, the cells were lysed and normalized to histone H4 level as indicator of the same number of cells. As indicated in Figure 1F, the PC12‐G2019S cells express a significant level of β3‐tubulin upon NGF treatment (with or without LEV) that is almost undetectable in the same cells treated with doxycycline. The simultaneous LEV administration in the doxycycline‐treated cells partially rescues the β3‐tubulin expression (Figure 1F).

### 3.2 Effect of LEV on DRD2 accumulation in Golgi areas due to LRRK2 G2019S expression

We have recently demonstrated that LRRK2 is implicated in the regulation of dopamine receptor trafficking. In particular, the expression of LRRK2 pathological mutant affects the dopamine receptor D2 (DRD2) localization,^7^ determining a significant receptor accumulation into the Golgi areas. Taking advantage of this experimental model, we have evaluated the LEV effect on the DRD2 localization in the presence of LRRK2 G2019S mutant. In a preliminary experiment, we have evaluated the LEV effect on DRD2 protein level and localization. No effect of 48 hours of LEV treatment was visible in SH‐SY5Y stably expressing DRD2 (SH‐SY5Y‐DRD2) (data not shown). Then, the SH‐SY5Y‐DRD2 was transduced by recombinant adenovirus expressing G2019S, treated or not with LEV and analysed 48 hours later. As previously reported,^7^ the cells expressing the G2019S show an important accumulation of DRD2 into the Golgi areas and this phenotype is significantly rescued by LEV treatment (Figure 2A‐B). The result is further confirmed by the analysis of DRD2 protein accumulation (Figure 2C‐D) as in Ref^7^. To further validate these results, we have analysed possible SV2A alteration by LEV treatment both at protein level (Western blot) and localization (immunofluorescence) without detecting any significant difference (data not shown).

## 4 DISCUSSION

The binding to SV2A protein is responsible for the antiepileptic action of LEV, a compound largely used in human therapy. Whether LEV binding is reducing or enhancing SV2A physiological function is largely unknown. Of note, the absence of SV2A protein in knockout animals results in the development of frequent seizures, suggesting an agonistic rather than an antagonistic effect of LEV on SV2A. Different lines of evidence indicate that both LRRK2 and SV2A are involved in common protein network^10^, ^11^ that modulate the vesicle trafficking/dynamics. Moreover, alteration in vesicle trafficking seems a common feature shared by different PD causative genes.^5^ Here, we show using three different experimental models (primary neurons, PC12 cells and SH‐SY5Y) that LEV is able to significantly revert the LRRK2 G2019S‐associated pathological effects. Interestingly, LEV attenuates rotenone‐induced toxicity in a rat model of PD,^13^ and in human, LEV treatment leads to an improvement in memory performance in patient with amnestic mild cognitive impairment (aMCI).^14^ In the past, LEV has been tested in the control of levodopa‐induced dyskinesia (LID) and, based on the conflicting results, for the International Parkinson and Movement Disorder Society the efficacy conclusion is ‘insufficient evidence’ although there are no safety concerns.^15^ Importantly, different side effects have been associated with LEV treatment or discontinuation. Among them, drowsiness, weakness, infection, loss of appetite and changes in behaviour and mood, including increased risk of suicide, are the most frequent. Some of these adverse effects were partially reduced by brivaracetam a new SV2A ligand with higher affinity compared with LEV. Although our research is far from the clinical application, our data support the idea that LEV repositioning may represent a valuable neuroprotective compound for PD, especially for PARK8 patients, that deserves future investigations.

## CONFLICT OF INTEREST

The authors confirm that there are no conflicts of interest.

## AUTHORS' CONTRIBUTIONS

RM, BA, FM and PG carried out the experiments. IC wrote the manuscript with support from CC and GE. IC, CC, GE and PG conceived and planned the experiments. SP and GM contributed to sample preparation. RM, B.A, IC, CC., GE and PG contributed to the interpretation of the results. All authors provided critical feedback and helped shape the research, analysis and manuscript.
