# Supplementary figures and images for: Levetiracetam treatment ameliorates LRRK2 pathological mutant phenotype
Source: J Cell Mol Med. 2019 Sep 27;23(12):8505–10. doi: 10.1111/jcmm.14674 (PMC6850958; doi:10.1111/jcmm.14674)

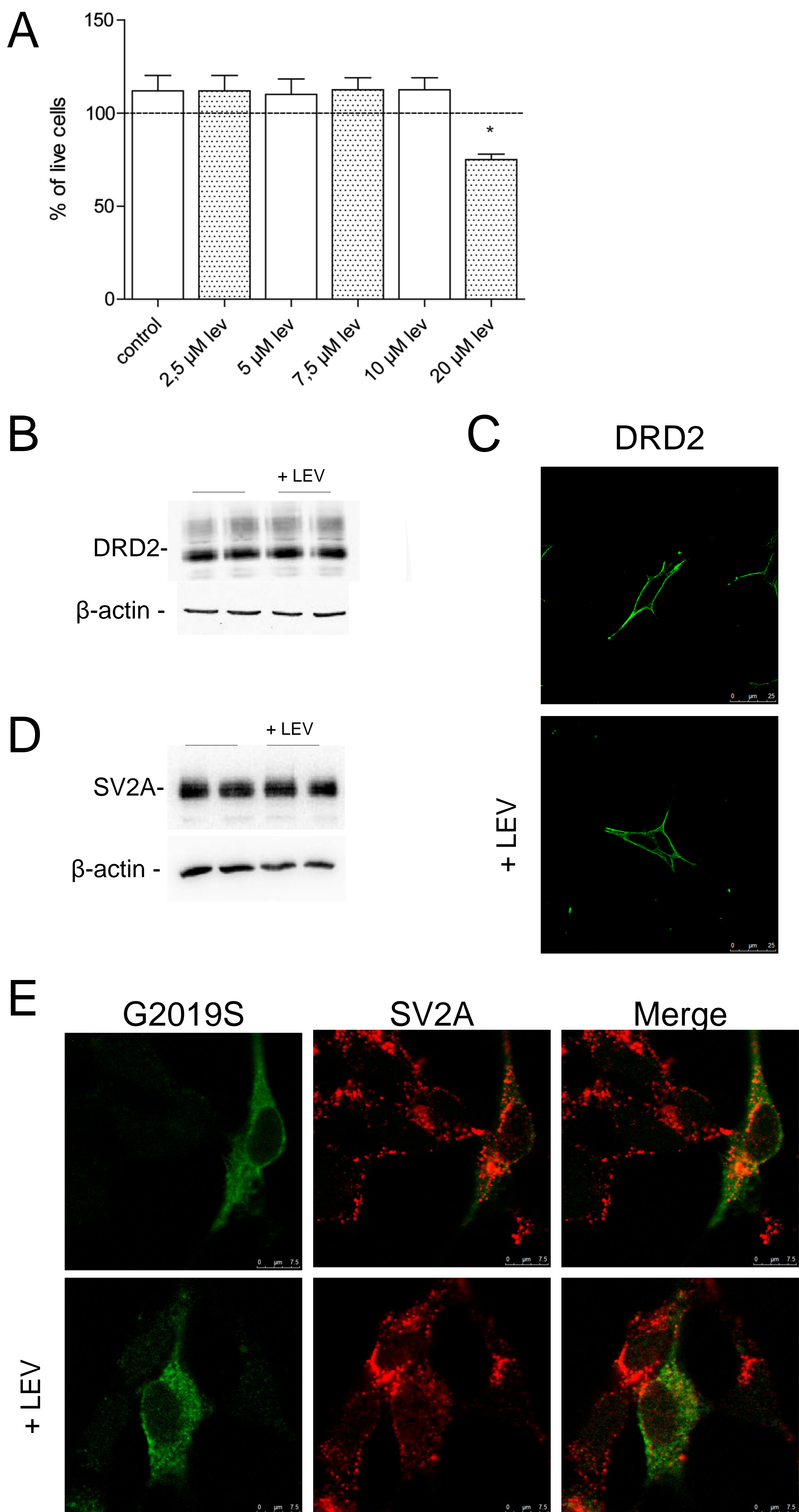

Figure S1

Supplement: Supplementary file 1 [file JCMM-23-8505-s001.pdf]
