# Supplementary material for: Levetiracetam treatment ameliorates LRRK2 pathological mutant phenotype
Source: J Cell Mol Med. 2019 Sep 27;23(12):8505–10. doi: 10.1111/jcmm.14674 (PMC6850958; doi:10.1111/jcmm.14674)
Supplement: Supplementary file 2 [file JCMM-23-8505-s002.docx]

**Supplementary Figure 1.** Analysis of LEV effect on primary neuron toxicity and on DRD2 or SV2A protein level and localization. (A) Primary neurons were treated for 48 hours with the indicated LEV concentrations and cell viability was analyzed by MTS assay (B) SH-SY5Y cells stably expressing Flag-tagged DRD2 were treated or not for 48h with LEV. The cells were lysed and incubated with different primary antibodies (anti-Flag for DRD2 and anti- β-actin as controls for equal loading of samples). (C) Cells treated as previously described were fixed and incubated with the anti-Flag for DRD2 staining. (D) SH-SY5Y cells were treated or not for 48h with LEV. The cells were lysed and incubated with different primary antibodies (anti-SV2A and anti-β-actin as controls for equal loading of samples). (E) SH-SY5Y cells were transfected with LRRK2 G2019S and treated or not for 48 hours with LEV. The cells were fixed and incubated with the anti-SV2A or anti-Myc antibody for LRRK2. In the same microscope field there are cells transfected or not by LRRK2 G2019S.
